# Automatic Extraction of Offshore Platforms in Single SAR Images Based on a Dual-Step-Modified Model

**DOI:** 10.3390/s19020231

**Published:** 2019-01-09

**Authors:** Jing Zhang, Qi Wang, Fenzhen Su

**Affiliations:** 1 Faculty of Geomatics, Lanzhou Jiaotong University, Lanzhou 730070, China; zhangj@lreis.ac.cn; 2 State Key Laboratory of Resources and Environmental Information System, Institute of Geographical Sciences and Natural Resources Research, CAS, Beijing 100101, China; wangqi@lreis.ac.cn; 3 Gansu Provincial Engineering Laboratory for National Geographic State Monitoring, 88 Anning Rd., Lanzhou 730070, China

**Keywords:** dual-step-modified model, two-parameter CFAR detection, Hough transform, single SAR image, offshore platform

## Abstract

The quantity and location of offshore platforms are of great significance for marine oil spill monitoring and offshore oil-gas development. In the past, multiphase medium- and low-resolution optical or radar images have been used to remove the interference of ship targets based on the static position of a platform to extract the offshore platform, resulting in large demands and high image data costs. According to the difference in shape between offshore platforms (not elongated) and ships (elongated shapes) in SAR (synthetic aperture radar) images, this paper proposes an automatic extraction method for offshore platforms in single SAR images based on a dual-step-modified model. First, the two-parameter CFAR (constant false alarm rate) algorithm was used to detect the possible offshore platform targets; then, the Hough transform was introduced to detect and eliminate ship targets with linear structures. Finally, the final offshore platform was obtained. Experiments were carried out in four study areas in the Beibu Gulf basin and the Pearl River estuary basin in the northern South China Sea. The results show that the method has a good extraction effect in the above research area, and the extraction accuracy rate of offshore platforms is 86.75%. A single SAR image can obtain satisfactory extraction results, which greatly saves on image data cost.

## 1. Introduction

With the evolution of offshore drilling technology, the exploitation of offshore oil-gas resources by countries around the world has increased, and the number of offshore platforms has also increased each year. To some extent, the locations and quantities of offshore platforms reflect the strength and capability of offshore oil-gas development in a country. At the same time, knowledge on the distribution of offshore platforms is of great value for the detection of oil spills. Therefore, obtaining the quantity and spatial distribution information of offshore platforms has important practical significance for the sustainable development and utilization of marine oil-gas resources and marine environmental monitoring.

It is difficult and costly to obtain the distribution of offshore platforms through field observations. Therefore, the use of remote sensing images for the extraction of large-scale offshore platforms has become an economic and efficient method to do so. Currently, many researchers have used optical and radar images to conduct offshore platform extraction. Liu Yongxue et al. (2016) adopted time-series and multi-refinement strategies to extract offshore platforms using landsat-8 Operational Land Imager (OLI) imagery with relatively complete datasets that largely overcome the influence of the maritime weather environment in the optical imagery and ship interference on the results. However, the imaging range of landsat-8 OLI imagery is only over land and offshore areas; therefore, this method is limited to the detection of offshore platforms near the coast [1]. Li Qiang et al. (2017) used the thermal infrared band of Visible-Infrared Imager-Radiometer Suite (VIIRS) data to detect exhaust fumes burning from offshore platforms. Regarding ship interference with the results, based on the static position characteristics of the offshore platform, a two-phase image was used to eliminate interference. However, the image was seriously marred by fire, clouds and other stray light, and it is still necessary to propose a fire and cloud detection algorithm to eliminate their influences [2]. The above studies indicate that the use of multiphase optical image extraction for offshore platforms is greatly affected by weather changes over the ocean, and it is difficult to achieve macro, long-term, continuous, and dynamic ocean observations [3].

As a microwave remote sensing system, SAR (synthetic aperture radar) is independent of light, weather and other conditions. SAR has the advantages of all-day time and all-weather and wide coverage and high resolution, and it also has a certain ground penetrating ability [4,5,6]. Casadio, et al. (2012) used two types of instruments, the SAR and the along-track scanning radiometer (ATSR), to extract offshore platforms. The SAR is used to unambiguously individualize the positions of offshore platforms with an accuracy of a few hundred meters, while the ATSR provides night-time radiance values that are used to individualize flaring events at the retrieved SAR locations. Using time-series imagery, an offshore platform was identified based on its fixed position to remove ships [7]. Wang Jiasheng et al. (2013) extracted an offshore platform from Environmental Satellite Advanced SAR (ENVISAT ASAR) images with similar imaging times for two scenes based on the two-parameter constant false alarm rate (CFAR) algorithm and compared the results of the two-phase extraction to remove the ship targets, and the results were relatively ideal. However, it is still possible that the ship did not move within a certain period of time; therefore, more images were taken for comparison to eliminate ship interference [8]. Since an offshore platform has the same materials and similar reflection characteristics as a ship, based on the fixed position of the offshore platform, two-phase or even multiphase images have been used to eliminate the interference of moving ship targets. However, using multiphase images to eliminate ship interference faces high image cost and low data processing efficiency. At the same time, due to the influence of image revisiting, it is sometimes difficult to obtain multiple images in the same region, which affects the accuracy of the target extraction of offshore platforms.

Focusing on the above problems, this paper proposes a method for extracting offshore platforms using a single SAR image based on a dual-step-modified model. First, a two-parameter CFAR algorithm is used to detect possible offshore platform targets. Then, according to the difference in the shape of the offshore platform and ship in the SAR image, ships with linear structures are detected and removed by the Hough transform; finally, the offshore platforms are output. Four study areas in the Beibu Gulf basin and the Pearl River estuary basin in the South China Sea are selected for the offshore platform extraction experiments and, using ground truth data for the precision evaluation and analysis of the extracted results, the existing results from the study of factors affecting the accuracy and shortcomings of the method are discussed and pointed out in the following section of the study.

## 2. Automatic Extraction of Offshore Platforms Based on a Dual-Step-Modified Model

Serious interference from ship targets exists in offshore platform extraction. This model is constructed from two aspects: the detection of the possible offshore platform targets and the elimination of ships. The two-parameter CFAR algorithm is capable of adapting to background clutter changes, setting the window size according to the size of the offshore platform, and performing threshold detection on each pixel to obtain a possible offshore platform target. According to the difference in the shape between a platform and a ship in a SAR image, the Hough transform is used to detect and eliminate a ship target with a linear structure. The remaining targets are offshore platforms. In this way, the goal of extracting an offshore platform using a single SAR image is achieved.

### 2.1. Possible Target Detection

At present, the algorithms used for offshore target detection include the Otsu algorithm [9,10,11], the Kapur, Sahoo and Wong (KSW) entropy method [12,13,14], and the CFAR algorithm [15,16,17]. Among these, CFAR is a relatively developed target detection method. Fin and Johnson (1968) proposed the cell-averaging CFAR (CA-CFAR) algorithm, which is suitable for single-objective scenes with a relatively homogeneous background area [13,18,19,20]. To solve the problem of multiple clutter occurrences in the background window, Trunk, G.V. (1978) proposed the detection method of the smallest option CFAR (SO-CFAR) algorithm and the greatest option CFAR (GO-CFAR) algorithm, which are effective for scenes where some strong clutter exists in the window, but the detection performance is reduced when clutter edge exists in the window [19,21,22,23]. Complex sea conditions and imaging characteristics make the background clutter of an SAR image inhomogeneous, thus generating a large number of false alarm targets; therefore, the results of global threshold automatic detection are not reliable [24,25]. It is necessary to apply a detection algorithm with local adaptive ability to perform target detection while maintaining a CFAR [26]. The two-parameter CFAR algorithm is a local adaptive algorithm and has been widely used in automatic target detection. It assumes that the background clutter is Gaussian and uses local windows to accommodate changes in background clutter. The size of the partial window can be set according to requirements and is easy to implement [27,28,29].

**Figure 1:**
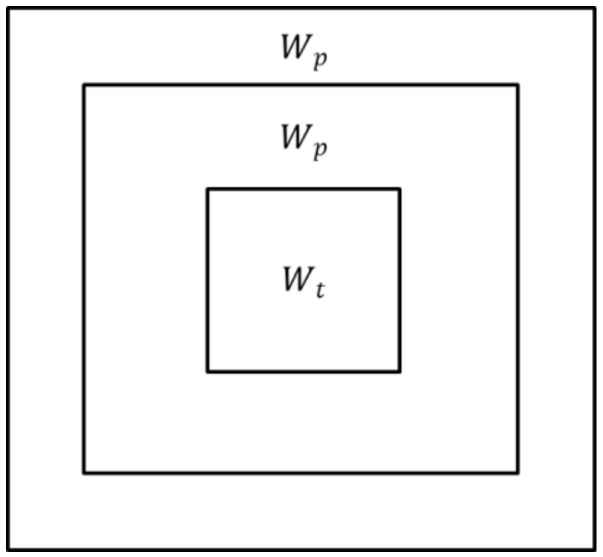
A schematic diagram of the two-parameter constant false alarm rate (CFAR) detection window, where $W_{b}$ represents the background window, $W_{p}$ represents the protection window, and $W_{t}$ represents the target window.

When implementing the two-parameter CFAR algorithm to detect the offshore platform, there are three detection windows: the target window $W_{t}$, the protection window $W_{p}$, and the background window $W_{b}$, as shown in Figure 1. The protection window ensures that the target pixels do not leak into the background window, which represents the statistical information of the sea clutter background. The target window is mainly used for the detection of offshore platform targets. The general target window is the size of the smallest offshore platform target in the detection image, the protection window is the size of the largest offshore platform target, and the background window is twice the size of the protection window [8]. The criteria for the two-parameter CFAR algorithm for target detection are [8,30]:

(1) $$\mu_{t}>\mu_{b}+\sigma_{b}\;t$$

In the above formula, $\mu_{t}$ represents the mean intensity in the target window, $\mu_{b}$ represents the background mean, $\sigma_{b}$ represents the standard deviation of the background, and *t* represents the control coefficient of the false alarm rate.

The control coefficient of the false alarm rate t has a significant influence on the detection results. When *t* increases, the number of false alarms decreases, and the number of false negative increases. In contrast, when *t* decreases, the number of false negative decreases, and the number of false alarms increases. Therefore, it is necessary to adjust *t* repeatedly to achieve the best balance between the test probability and false alarm rate. Generally, the empirical value is 4.5 < *t* < 7.5 [8].

When searching for detections in the entire SAR image, the above three windows slide in the image according to a certain step size (generally the same as the target window) and, one by one, they determine whether the point in the target window is the target point.

### 2.2. Ship Target Detection and Elimination

**Figure 2:**
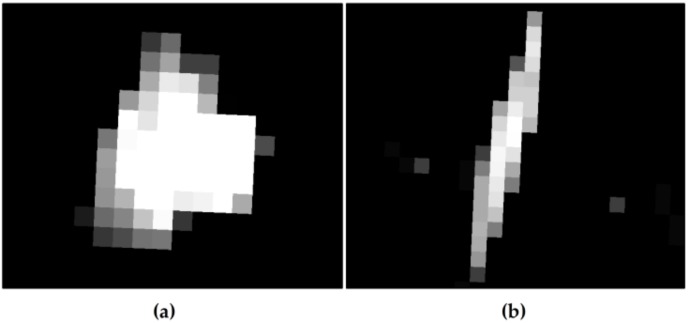
Shape results of the offshore platform and ship in the GF-3 synthetic aperture radar (SAR) image. (**a**) shows the offshore platform, and (**b**) shows the ship.

Considering that the possible targets detected by the two-parameter CFAR algorithm include offshore platforms and ships, it is necessary to further distinguish them and eliminate ship targets. The shape of ships and offshore platforms are different in an SAR image. Ships mostly appear in elongated shapes, while platforms are mostly not elongated, as shown in Figure 2. Therefore, based on the shape difference between the two, the Hough transform is introduced to detect ship targets to eliminate ship target interference.

The Hough transform is a transformation algorithm between coordinate space and parameter space that was first proposed by Hough in 1962 and is often used for line identification. Its basic idea is to transform the linear detection in an image space to the parametric space to find the peak value that satisfies the linear characteristics [31,32,33].

The principle of line detection based on Hough is as follows: in Cartesian coordinates, a line can be represented by the equation *y* = *kx* + *b*. For a certain point (*x*_0_, *y*_0_) on the line where *b* = *y*_0_ − *kx*_0_, it can also become a line with −*x*_0_ as its slope and *y*_0_ as its intercept. The original Cartesian coordinate system becomes the (*k* − *b*) parameter coordinate system, and then all points on the original line become multiple lines in the (*k* − *b*) coordinate system. Therefore, the coordinate values *k* and *b*, representing the plurality of the straight-line common intersection points in the parameter space, are the parameters of the line equation to be detected. There are several intersections that represent several lines in the Cartesian coordinate system.

**Figure 3:**
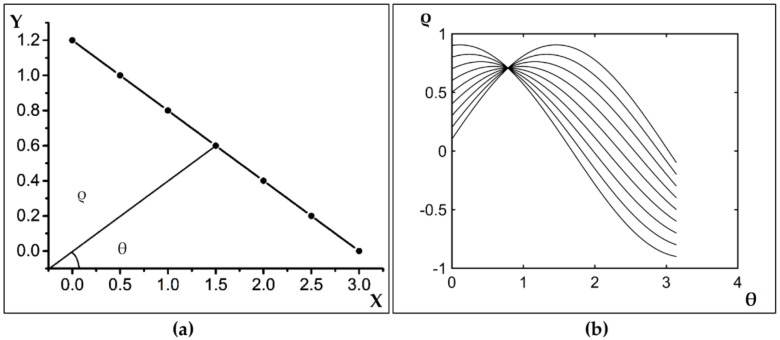
A schematic diagram of the Hough transform, where (**a**) shows a schematic diagram of the Cartesian coordinates, and (**b**) shows the parametric space curve diagram.

However, when the line is perpendicular to the *x*-axis (i.e., when the slope does not exist), the point-slope form cannot be expressed, and the polar coordinate system can solve this problem. Any line in the plane is represented by a polar coordinate equation, which can be determined by two parameters: ρ and θ. For any point in the image space, the functional relationship is:

(2) ρ=x cosθ+y sinθ

where θ∈[0,π], θ represents the angle between ρ and the positive *x*-axis, and ρ represents the distance from the point to the line [34,35,36]. If the abovementioned transformation is performed on n points located on the same straight line *L*, n points of the original image space correspondingly obtain n sinusoids in the parameter space, and the curves intersect at one point, indicating that the points are on the same line, as shown in Figure 3 [37].

Therefore, the method of detecting the ship target by using the Hough transform is to map the image to the parameter space and construct the parameter space curve [38,39]. If all curves intersect at one point, all of the pixels constituting the possible target are on the same line, which is in line with the ship target. The linear feature is considered to be a ship, and it is not a ship target, which does not meet the characteristics of the abovementioned parameter space curve. This paper implements the detection of ship targets in remote sensing images based on the Hough transform principle in MATLAB. First, the following operations are performed using the correlation function in MATLAB. Therefore, the sobel operator is used to automatically select the threshold to binarize the image, thus achieving the purpose of edge detection. These edges are the continuous pixels that make up the ship’s target. Specifically, the Hough transform was performed by using the Hough function to obtain a Hough matrix. The target peak point in the Hough matrix was obtained by the Hough peaks function. The Hough lines function was used to obtain the ship target in the original binary image based on the results of the previous two steps. After many experiments, the threshold of the Hough peaks function was set to 0.3, the threshold of the ship consolidation in the Hough lines function was set to 20, and the minimum length threshold (MinLength) of the detected ship was set to 0.1.

### 2.3. Automatic Extraction Process of Offshore Platforms

**Figure 4:**
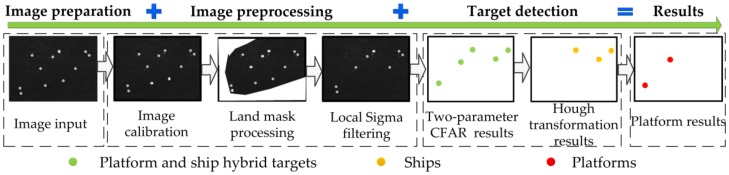
A schematic diagram of the automatic extraction process of an offshore platform.

The automatic extraction process of offshore platforms based on the dual-step-modified model proposed in this paper mainly includes four parts: SAR image preprocessing, target detection of possible offshore platforms, ship target detection and elimination, and offshore platform extraction. The details are as follows (Figure 4):

(1) First, SAR image preprocessing is carried out, including geometric and radiation corrections, study area image masking and image filtering;
(2) Then, the two-parameter CFAR algorithm is used to detect the possible target of offshore platforms after SAR image preprocessing, and the detection results include offshore platforms and ships;
(3) Furthermore, the Hough transform is performed to detect the ship targets;
(4) Finally, the detection results in steps (2) and (3) for the intersection with ship targets are eliminated, and the offshore platform targets are output.

## 3. Case Application

### 3.1. Study Areas and Datasets

#### 3.1.1. Study Areas

**Figure 5:**
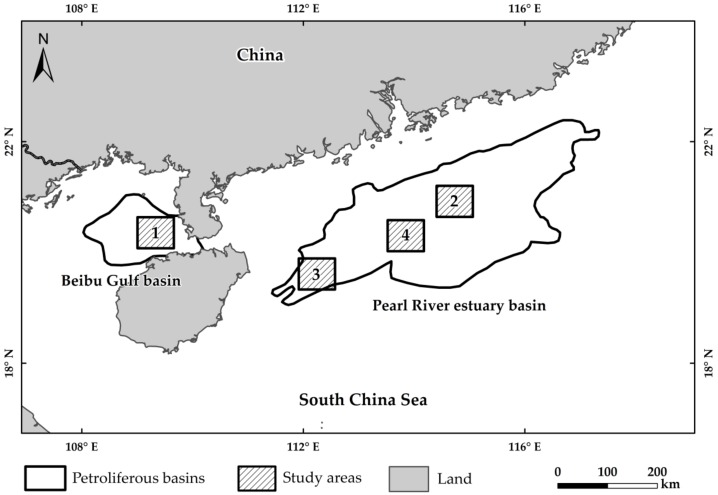
Diagram of the research locations. The gray square in the figure indicates the petroliferous basins, and numbers 1–4 represent regions in the selected study area where the offshore platforms are densely distributed.

The Beibu Gulf basin and the Pearl River estuary basin in the northern part of the South China Sea were selected as the study areas (19.83° N–21.011° N, 108.119° E–110.13° E; 19.46° N–21.99° N, 111.51° E–117.17° E) (Figure 5). These areas are the main oil-gas producing areas in China. The offshore platforms are built intensively, and the image coverage is relatively complete. Therefore, this region is selected for the study (Figure 5).

#### 3.1.2. Datasets

The data used in this study include image and auxiliary data. Image data were used to detect offshore platform targets and analyze the results. Auxiliary data were used to show the spatial distribution of offshore platforms and evaluate the extraction precision.

**Figure 6:**
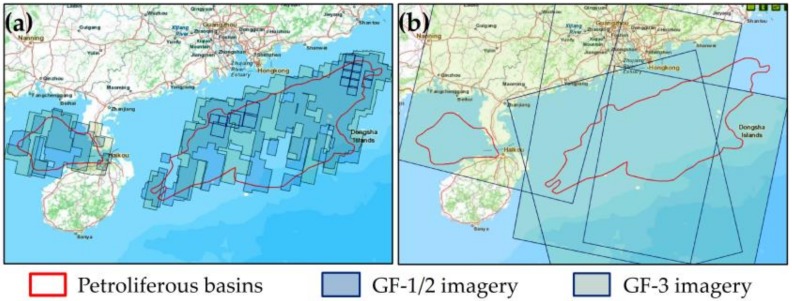
The image coverage of the study area. (**a**) shows the coverage of the GF-1/2 image and (**b**) shows the coverage of the GF-3 image.

Image data include high-resolution optical image GF-1/2 and medium-resolution radar image GF-3 SAR developed in China. Since 2013, China’s high-resolution Earth observation system has successfully launched six satellites. Among them, the GF-1-GF-3 satellites are loaded with sensors (e.g., particular matter sensor (PMS) and SAR), with a maximum spatial resolution of 1 m and a maximum temporal resolution of 1 d, that derive from the China Center for Resources Satellite Data and Application (http://www.creda.com/CN/) website. GF-3 has 11 imaging modes, of which the wide-mode wide ScanSAR (WSC) has a spatial resolution of 40 m and an image width of 500 km × 500 km, which satisfies the resolution and image coverage requirements for offshore platform detection. The backscattering intensity of the SAR image depends on the radar wave polarization mode. In general, the co-polarization (HH, VV) mode is more favorable for a target whose structure is parallel to the radar’s viewing direction, and cross polarization (HV, VH) better detects targets at a certain angle from the radar’s viewing angle [40,41]. The deck of the main structure of the offshore platform is generally located at a height of approximately 20 m above the water surface and forms a certain angle with the radar viewing direction; therefore, the GF-3 SAR HV polarization mode was selected. The GF-1/2 optical image was mainly used for the analysis of the extraction results. Considering the influence of clouds on imaging, imagery where the cloud cover is less than 20% was selected. The image temporal information and spatial coverage are shown in Table 1 and Table 2, and Figure 6.

Auxiliary data include (1) petroliferous basin data (refer to “Atlas of China’s petroliferous basins”); (2) vector data, such as national border lines, which are provided by the global administrative division website (http://www.nhjd.net/); and (3) ground truth data obtained from the surveys of offshore platforms conducted in May 2017, including the number and location of the offshore platforms (Table 3).

Before offshore platform detection, image data (GF-1/2 and GF-3) are subject to a series of calibration processes, including radiation calibration and geometric calibration, to eliminate the impact of the image itself on target detection. The image metadata were used to complete the calibration process [42]. Offshore platforms are mostly distributed in offshore areas far from the shore, and the types of near shore materials are complex, including a large number of artificial facilities made of metal, which have interference effects on the detection results. Therefore, the GF-3 SAR data used for offshore platform detection should establish a land mask to eliminate the interference of near shore and terrestrial information on the results. Since the SAR is a coherent system, speckle noise is an inherent phenomenon and requires speckle noise filtering [43,44]. The adaptive filter preserves the high-frequency information and details of the image while suppressing noise and mainly includes the Gamma filter, Kuan filter, and local Sigma filter. After repeating the experiments, the local Sigma filter preserves the high-frequency information and details of the image while suppressing noise [45,46,47], which has the greatest filtering effect. Therefore, the local Sigma filter was used to filter the GF-3 SAR image.

### 3.2. Precision Evaluation Method

To evaluate the ability of the proposed method to automatically extract the offshore platform, based on a ground truth data of offshore platform in the study areas, we define the accuracy rate (*a*), false negative rate (*n*), and false alarm rate (*f*) of the three precision indexes to analyze the automatic extraction precision. The ground truth results are the actual number of offshore platforms.

The accuracy rate (*a*) was used to measure the accuracy of automatic extraction of the offshore platform and is the ratio between the number of automatic extractions and the actual number. The formula is as follows:

*a* = *N_S_*/*S* × 100% (3)

where *S* represents the actual number of offshore platforms obtained by ground truth data and *N_S_* represents the number of offshore platforms that are automatically extracted correctly.

The false negative rate (*n*) indicates the proportion of the failed to find numbers in the automatic extraction of the offshore platform. The formula is as follows:

*n* = *N_n_*/*S* × 100% (4)

where *S* represents the real number of offshore platforms obtained by ground truth data. *N_n_* represents the number of offshore platforms that are false negative.

The false alarm rate (*f*) is used to measure the extent of the offshore platform that is misidentified, and the formula is as follows:

*f* = *N_f_*/*S* × 100%, (5)

where *S* represents the real number of offshore platforms obtained by ground truth data. *N_f_* represents the number of offshore platforms that automatically extract errors.

### 3.3. Comparison and Analysis with High-Resolution Imagery

To further compare and analyze the accuracy of the extraction results with high-resolution GF-1/2 imagery, the extracted offshore platform targets were positioned in high-resolution images. Every target (correct, error, and omitted) is verified in GF-1/2. The reasons for error and omission are analyzed as much as possible.

### 3.4. Results

#### 3.4.1. Extraction Results in the Study Areas

**Figure 7:**
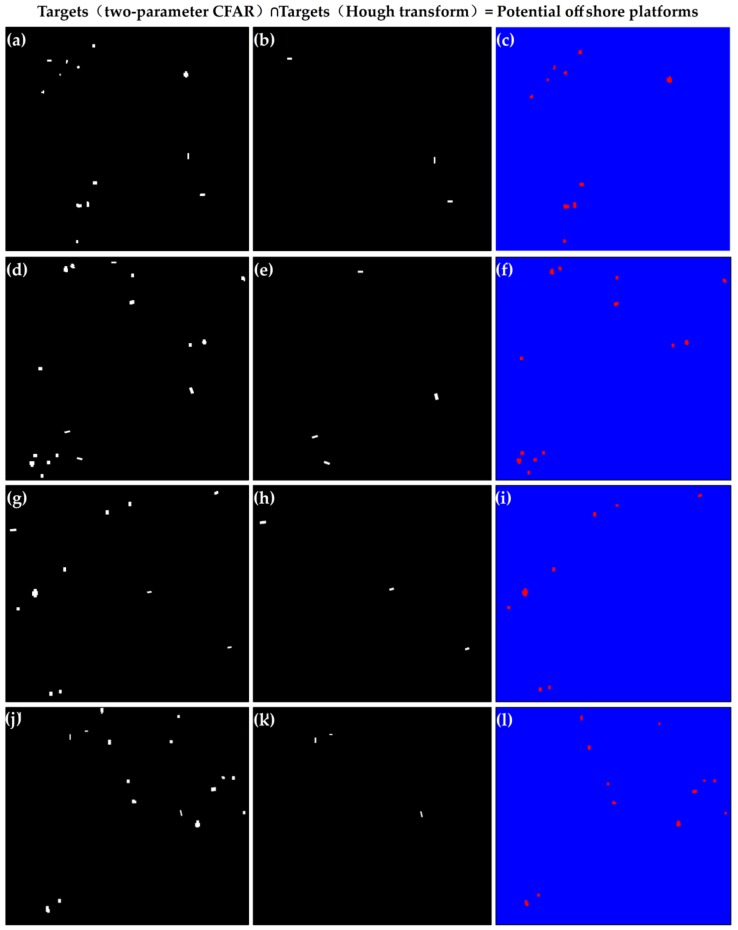
Results of the automatic extraction of offshore platforms in the four study areas, where (**a**–**c**) represent study area 1, (**d**–**f**) represent study area 2, (**g**–**i**) represent study area 3, and (**j**–**l**) represent study area 4. (**a**,**d**,**g**,**h**) show the two-parameter constant false alarm rate (CFAR) detection results of study areas 1–4, (**b**,**e**,**h**,**k**) show the Hough detection results of study areas 1-4, and (**c**,**f**,**i**,**l**) show the final potential offshore platform extraction results of study areas 1–4.

The offshore platform extraction method based on a dual-step-modified model was applied to 2 tiles of GF-3 SAR images covering the four selected study areas in the Beibu Gulf basin and the Pearl River estuary basin. The extraction results are as follows: a total of 43 potential offshore platforms in four study areas were extracted by this method. Among these, the results of the four study areas are shown in Figure 7. Study area 1 (Figure 7a–c) removed 3 ship targets and extracted 10 potential offshore platforms. In study area 2 (Figure 7d–f), after removing four ship targets, 13 potential offshore platform targets were finally obtained (Figure 7f). Three ship targets were detected and removed (Figure 7h), and a total of 8 potential offshore platforms were extracted in study area 3 (Figure 7i). Study area 4 shows that the two-parameter CFAR detected 16 possible targets in Figure 7j, removing three ships (Figure 7k), and a total of 12 potential offshore platform targets were extracted (Figure 7l). Specifically, the results are shown in Table 4.

#### 3.4.2. Precision Evaluation Results

According to the precision evaluation method, the extraction accuracy rate (*a*), false negative rate (*n*), and false alarm rate (*f*) of the four study areas were statistically analyzed based on the actual number of offshore platforms acquired by ground truth data. The results are shown in Table 5.

From Table 5, the application of the proposed method can achieve a single SAR image for the automatic extraction of the offshore platform, and the overall extraction accuracy is better. In the four study areas selected, only the second study area has poor results, and the other three study areas achieve approximately 90% accuracy. Finally, according to the average accuracy obtained in the four research areas, an accurate extraction rate of up to 86.75%, a false negative extraction rate of 12.25%, and an error extraction rate of 6.5% are achieved. The precision evaluation results indicate that the proposed method can satisfy the needs for practical application.

#### 3.4.3. Comparison and Analysis Results with High-Resolution Imagery

**Figure 8:**
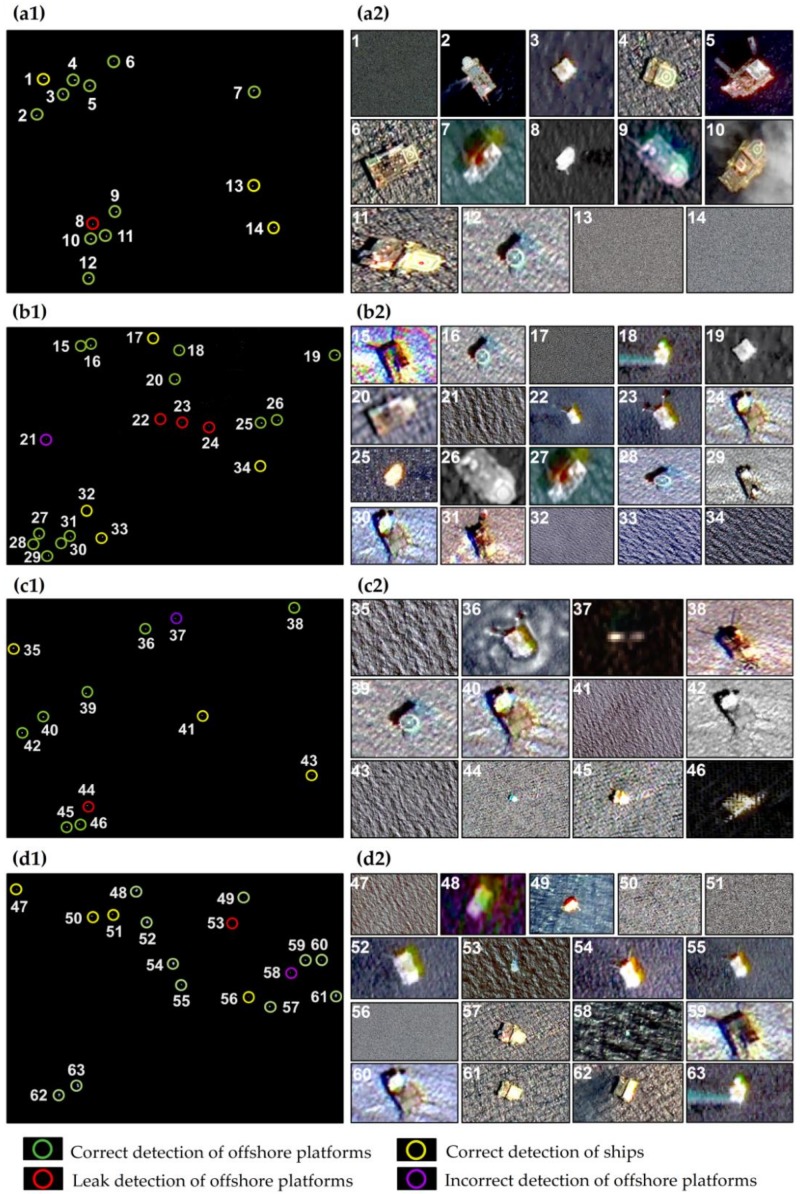
Comparison and analysis results with the GF-1/2 imagery. (**a1**–**d1**) show the detection results and ground truth data of study areas 1–4; (**a2**–**d2**) show the results of comparison and verification with GF-1/2 for study areas 1–4.

A total of 25 tiles of high-resolution GF-1/2 images were selected to compare and analyze the extraction results in the four study areas. Through careful visual discrimination, in study area 4 (Figure 8(a1,a2)), 10 offshore platforms were correctly detected, and the No. 8 offshore platform target was a false negative. The GF-1 image showed that the No. 8 offshore platform had a small size; therefore, the brightness in the SAR image was weak, which made the two-parameter CFAR algorithm undetected, resulting in false negative detection. The target Nos. 1, 13 and 14 were detected as three ships by the Hough transform, which were verified in the GF-1 and GF-2 images. In study area 2, 12 offshore platforms were correctly detected after removing 4 ship targets (Figure 8(b1,b2)). Target Nos. 22–24 were all shown as offshore platforms in the GF-1 image, but they were undetected. The analysis found that the sea clutter in the GF-3 SAR image in this area was more complex, which undoubtedly affected the detection threshold of the two-parameter CFAR algorithm, resulting in false negative detection. In addition, target No. 21 showed in the GF-2 image that there was no offshore platform or ship, which was also due to the influence of sea clutter and error detection of the offshore platform. In study area 3 (Figure 8(c1,c2)), target No. 37 was a ship in the GF-1 image, and the ship had stayed in this area through multiphase imagery. The ship was small, which does not meet the detection threshold of the Hough transform and is incorrectly detected for the offshore platform. Target No. 44 was actually an offshore platform in the GF-1 image. Due to its small size, it was not detected in the two-parameter CFAR algorithm, resulting in false negative detection. Finally, 7 offshore platforms were correctly detected after removing 3 ships in this study area. In study area 4, a total of 11 offshore platforms were correctly detected. Target No. 53 was an offshore platform in the GF-1 image. According to the ground truth data, this offshore platform had no oil–gas production operations in the last half year due to aging equipment; therefore, it was undetected by the two-parameter CFAR algorithm. Target No. 58 was a lighthouse in the sea in the GF-1 image, which was similar to the imaging characteristics of the offshore platform in the GF-3 SAR image; therefore, it was incorrectly detected as an offshore platform (Figure 8(d1,d2)).

### 3.5. Parameters Used in the Automatic Extraction Method of Offshore Platforms

**Figure 9:**
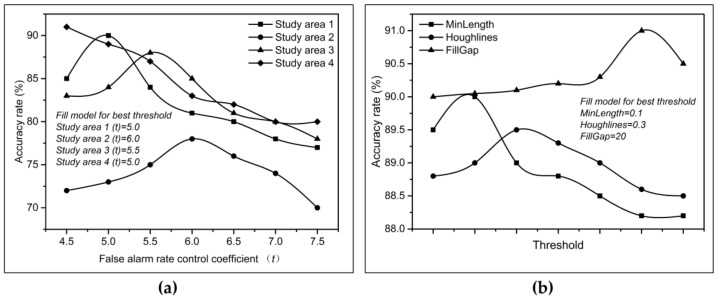
Optimal parameter comparison graph of the two-step correction model, where (**a**) shows the optimal detection threshold of the two-parameter constant false alarm rate (CFAR) algorithm in the four study areas and (**b**) shows the optimal detection threshold of the Hough transform in this paper.

The dual-step-modified model was applied to the single SAR images covering the Beibu Gulf basin and Pearl River estuary basin; the parameters applied to the method are shown in Table 6. The results of the parameter comparison between the two-parameter CFAR detection method and the Hough transform method of the dual-step-modified model are shown in Figure 9. Finally, the optimal threshold is selected for the above offshore platform extraction.

## 4. Discussion

### 4.1. The Effects of the Parameters on the Extraction Results

When using the two-parameter CFAR algorithm for offshore platform extraction, it is necessary to determine the size of the detection window and the value of the false alarm rate control coefficient *t*. The former can be determined by the size of the offshore platform to be detected and the image resolution used, while the latter needs to be adjusted according to the formula $\frac{\mu_{t}-\mu_{b}}{\sigma_{b}}>t$ to determine the final value of *t*, where $\mu_{t}$ represents the mean intensity in the target window, $\mu_{b}$ represents the background mean, and $\sigma_{b}$ represents the background standard deviation. Therefore, the value of *t* changes according to the change in the three values in the formula; the value of *t* needs to be adjusted repeatedly and has certain subjectivity.

### 4.2. Influence of Ocean Background Clutter on Target Detection

**Figure 10:**
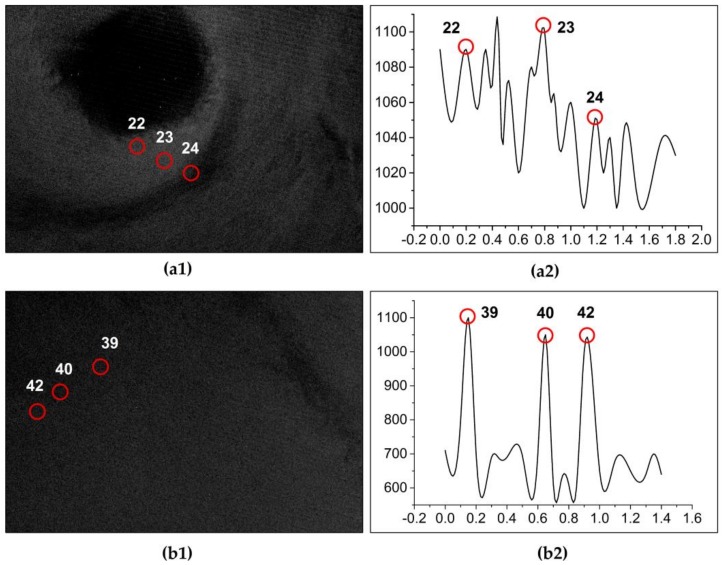
Image background histogram of the comparison results. (**a1**,**b1**) show the inhomogeneous and more homogenous background clutter of the synthetic aperture radar (SAR) image, respectively. (**a2**,**b2**) correspond to its background histogram.

For the detection of a possible offshore platform by using the two-parameter CFAR algorithm, it is necessary to calculate the background mean of each detection window and the background standard deviation of the image to determine the value of the false alarm rate control coefficient *t*. When the sea state in the image is more complicated, the brightness contrast between the target and background is small. In addition, the clutter strength in the detection window is large, which makes the modeling ability of the two-parameter CFAR algorithm decrease, raising the threshold. The accuracy of the background clutter model is directly related to the performance of the target detection, resulting in weaker target brightness. For example, the Nos. 22–24 platforms failed detection because the clutter in the SAR image background in study area 2 was inhomogeneous (Figure 10(a1)). In study area 3, only one offshore platform was omitted due to the more homogenous background clutter of the image (Figure 10(b1)). An image histogram of the different clutter backgrounds is shown in Figure 10.

### 4.3. Applicability Analysis of the Offshore Platform Automatic Extraction Method Based on the Dual-Step-Modified Model

The automatic extraction method of an offshore platform based on a dual-step-modified model aims to eliminate ship interference and extract an offshore platform by using a single-scene SAR image. The principle of the method is to utilize the difference in shape between the offshore platform and the ship in the medium-resolution SAR image, where the offshore platform is not elongated, and the ship is an elongated shape. The target with linear structural features in the SAR image is detected and eliminated by the Hough transform, and the remaining possible targets represent the final offshore platform. Therefore, the method has certain requirements regarding the spatial resolution of the SAR image. Taking the GF-3 image as an example, when the image spatial resolution is higher, the image width also decreases; the vastness of the ocean requires substantial image coverage, which increases image costs. Therefore, this method is not suitable for high-resolution SAR images. When the image resolution is lower, one pixel in the SAR image is one or two offshore platforms. At this time, the difference in shape between the offshore platform and the ship cannot be distinguished, and the Hough transform is meaningless in this model; therefore, the method is not applicable to low-resolution images. In this paper, the SAR image with a resolution of 40 m is easy to distinguish between the offshore platform and the ship target shape; thus, the Hough transform can be detected and eliminate the ship target. Therefore, this method is applicable to moderate-resolution images; at this time, this method can realize single SAR images to extract offshore platforms and save on image costs.

### 4.4. Limitations of the Hough Transform

**Figure 11:**
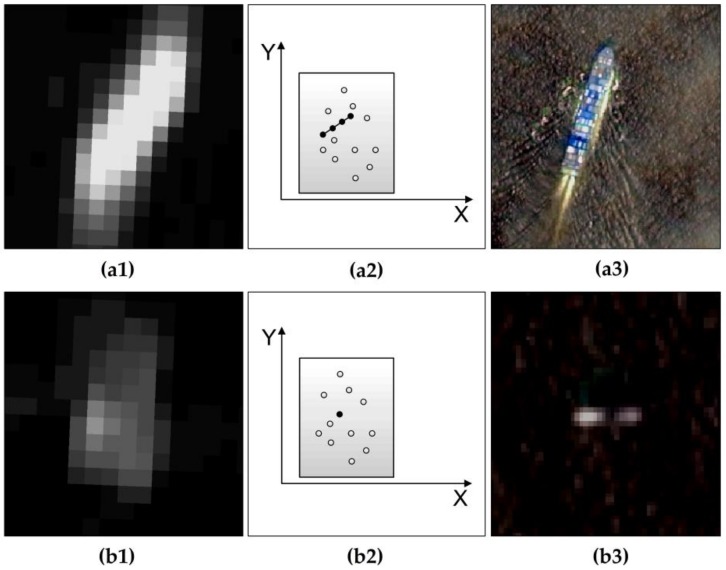
Influencing results of dim targets on the Hough transform. (**a1**,**b1**) are images of ships a and b in GF-3, respectively; (**a2**,**b2**) represent expressions of the pixel coordinate point sets of ships a and b in Hough space, respectively; (**a3**,**b3**) are images of ships a and b in GF-1/2, respectively.

In the process of using the Hough transform to detect and eliminate ships, the pixel set that constitutes the target satisfies the linear structure and is considered the ship target. Usually, the pixel set is ≥3 pixels, which is determined by the ship length and image resolution. If the size of the ship target is small, the target is missed, or an error is detected. The ships with smaller dimensions have weaker echoes and relatively weak brightness features in SAR images. The measurement of the Hough transform for a dim target is a pixel point in the echo data (Figure 11(d2)); therefore, it is impossible to form a line connecting several points in the radar echo data (Figure 11(c2)), and it cannot be detected. For example, ship target No. 37 in study area 3 is caused by the above reasons (Figure 8).

### 4.5. Applicability Analysis of Extracting Line Features in SAR Images Using Hough Transform

In the detection of ship targets using Hough transform, the SAR image is multiplicative speckle noise, and the optical image is additive noise, so the multiplicative noise of the SAR image will amplify the difference between the high reflection area and the low reflection area. Our research area is located in the ocean. The artificial targets such as offshore platforms and ships in the ocean differ greatly from the ocean background, while the difference between the ocean backgrounds is small. Therefore, for extracting ship targets at sea, multiplicative noise and additive noise have little effect on the detection results of the target.

Because of this, it is feasible to use the Sobel operator for binarization of the SAR image before the Hough transform to better detect the edge. The advantage of the Sobel operator is that the calculation speed is fast, because it only uses the template in two directions, only the edges in both the horizontal and vertical directions can be detected, and the shape of the ship target is simple, in the form of a line segment, so it has an ideal detection effect.

## 5. Conclusions

Timely and accurate information on the development of offshore platforms can provide a reference for countries to formulate marine development strategies and support decision-making for the management of marine environments. It is necessary to automatically extract offshore platforms from remote sensing images. However, both optical and radar images use two-phase or multiphase images to detect offshore platforms based on the invariant characteristics of offshore platform positions to eliminate ship interference, resulting in high image cost and higher requirements for image coverage and revisiting periods. An automatic extraction method for offshore platforms in a single SAR image based on a dual-step-modified model is proposed. According to the difference in shape between offshore platforms (not elongated) and ships (elongated shapes) in an SAR image, the model introduces the Hough transform to detect and eliminate the ship target to realize the purpose of offshore platform extraction by using a single SAR image.

From the results of the precision evaluation, the average accuracy rate of the offshore platform extraction in this method is 86.75%, the average false negative rate is 12.25%, and the false alarm rate is 6.5%. The extraction accuracy meets the needs of practical applications. This method can detect offshore platforms by using a single SAR image, which greatly saves the image data cost and effectively compensates for the deficiency in image coverage.

Although some progress has been made in extracting offshore platforms by using a single SAR image, there are still some disadvantages in the extraction accuracy. Further research will be carried out on enhancing the modeling ability of a complex background with sea clutter, the automatic determination of the detection threshold, and how to balance the insensitivity of the Hough transform with weak local pixels and robustness to random noise.

## Figures and Tables

**Table 1:** The GF-1/2 remote sensing image data (PMS = particular matter sensor).

No.	Satellite	Sensor	Resolution (/m)	Time	Cloud Cover (%)
1	GF-1	PMS	2	2017/6/12	5
2	GF-1	PMS	2	2017/7/2	7
3	GF-1	PMS	2	2017/7/24	6
4	GF-1	PMS	2	2017/8/9	3
5	GF-1	PMS	2	2017/8/26	3
6	GF-1	PMS	2	2017/9/2	0
7	GF-1	PMS	2	2017/9/15	0
8	GF-1	PMS	2	2017/9/23	9
9	GF-1	PMS	2	2017/9/23	8
10	GF-1	PMS	2	2017/9/23	11
11	GF-1	PMS	2	2017/9/26	11
12	GF-1	PMS	2	2017/10/19	13
13	GF-1	PMS	2	2017/10/19	0
14	GF-1	PMS	2	2017/10/22	0
15	GF-1	PMS	2	2017/10/22	9
16	GF-1	PMS	2	2017/10/22	15
17	GF-2	PMS	1	2017/10/23	12
18	GF-2	PMS	1	2017/10/23	3
19	GF-2	PMS	1	2017/11/5	1
20	GF-2	PMS	1	2017/11/5	0
21	GF-2	PMS	1	2017/11/17	7
22	GF-2	PMS	1	2017/11/26	9
23	GF-2	PMS	1	2017/11/27	13
24	GF-2	PMS	1	2017/11/27	8
25	GF-2	PMS	1	2017/12/1	5

**Table 2:** GF-3 remote sensing image data (SAR = synthetic aperture radar).

No.	Satellite	Sensor	Image Mode	Resolution (/m)	Pixel Spacing (/m)	Polarization Mode	Time
1	GF-3	SAR	WSC	40	40	HV	2017/9/22
2	GF-3	SAR	WSC	40	40	HV	2017/9/22

**Table 3:** Ground truth data of the offshore platforms.

Petroliferous Basins	Study Area	Numbers
Beibu Gulf basin	Study Area 1	11
Pearl River estuary basin	Study Area 2	14
	Study Area 3	8
	Study Area 4	12

**Table 4:** Extraction results in the study areas.

Study Area	Two-Parameter Constant False Alarm Rate (CFAR) Detection Results	Hough Transform Detection Results	Potential Offshore Platforms
Study area 1	13	3	10
Study area 2	17	4	13
Study area 3	11	3	8
Study area 4	16	3	12

**Table 5:** Precision evaluation results of the automatic extraction of offshore platforms.

	Study Area 1	Study Area 2	Study Area 3	Study Area 4	Mean
Actual number (*S*)	11	14	8	12	-
Total extraction (*N*)	10	13	8	12	-
Accurate rate (*a*)	90%	78%	88%	91%	86.75%
False negative rate (*n*)	9%	21%	11%	8%	12.25%
false alarm rate (*f*)	0	7%	11%	8%	6.5%

**Table 6:** Parameters used in the automatic extraction method of offshore platforms.

Categories	Parameters
Image preparation	2 images in GF-3 SAR covering four study areas (resolution = 40 m)
Image preprocessing	Calibration: backscattering coefficient and sensor calibration data are acquired from the metadata file
	Land mask: data over the extent of petroliferous basins provided by the “Atlas of China’s Petroliferous Basins”
	Local Sigma filtering: filter size: 3 × 3 (σ = 1)
Target detection	Detection threshold of the two-parameter constant false alarm rate (CFAR): *t* = 5.0 (study area 1), *t* = 6.0 (study area 2), *t* = 5.5 (study area 3), and *t* = 5.0 (study area 4) Detection parameters of the Hough transform: Hough peaks = 0.3; Hough lines: FillGap = 20; MinLength = 0.1
